# Experiments and Observations on Mesmerism

**Published:** 1829-09

**Authors:** Richard Chenevix


### MESMERISM.

*Experiments and Observations on Mesmerism.* By RICHARD CHENEVIX, Esq. F.R. and E S., M.R.I.A., &c. (4th Article.)

It is the duty of a conscientious narrator to relate, with equal frankness, every portion of his story; to give the testimony of all who depose against his doctrines, as well as in their favor; to record failure no less than success. In the present instance, however, this duty may be dispensed with, on account of the details into which it would lead me; and having, in a former article, premised that not more than one patient in six slept, or was sufficiently susceptible of the mesmeric influence to manifest convincing phenomena, I may spare my readers an enumeration of many experiments: telling them merely that, before I met with cases worthy of being submitted to the witnesses whose evidence I am going to adduce, I encountered many disappointments. Such is the fate of all who make researches upon mesmerism in its present state; and the happiest practical addition which could be made to the science now, would be some certain indication of mesmeric susceptibility, as nearly five-sixths of the labour would be abridged to all who could produce prompt and convincing results

Being in Dublin on the 26th of March, 1829, I made some experiments at the Hospital of Incurables there, by permission and in presence of Dr. Croker, physician to the establishment. The first six patients exhibited no effects; at least, none which could convince an inexperienced observer. The seventh patient was *Mary Glynne,* a severe case of vomiting, originally caused by an injury. She was obliged to have the nurse and a crutch to enable her to come down stairs, as she said she could not come down without their assistance. She could, however, with the help of her crutch alone just walk from her bed to the fireside in her own ward. For the last two months she had excruciating pains in her left arm, which Dr. Croker feared was threatened with paralysis. I had never seen this woman before, and now only in the presence of Dr. C. I desired her to sit down, and, without any conversation whatever, mesmerised her for thirty minutes. She then, of her own accord, said that she thought herself better, and believed she could walk. She did so. I made her sit down again, and in fifteen minutes more she had *clignotements* of her eyelids, complained that she was too warm, and got up again to walk. This time she used no crutch, and declared (what, indeed, was notorious in the hospital,) that for two months she had not been able to do as much. She was mesmerised again immediately, and, after about sixty minutes, got up again, and walked quite well. The nurse and attendants, when called into the room, expressed the utmost astonishment at seeing her pace along without the least unevenness in her gait; and she went up stairs to her ward without any assistance whatever, leaving even her crutch in the room where she had been mesmerised.

Second; March 27th.—She said she had slept better last night than she had done for a long time, and continues to walk quite well. She complained of a stinging pain in head and hand; has long been unable to put her left hand behind her back. No effect was produced on it by mesmerism; neither was any effect apparent upon her this day.

Third; March 28th.—Continues to walk perfectly well, insomuch that two medical students, who had not seen her till now, could hardly credit that, but forty-eight hours before, she could walk only with a crutch, and that very badly. She did not sleep so well last night, on account of an eruption on her head, caused, as it was supposed, by the use of nitric acid. She felt heavy and drowsy today during the operation. Her left arm is not getting better. Saw her no more after this day.

The improvement produced in this case, though so much yet remained undone, was most striking, and made a forcible impression upon all who were present, Dr. Croker and three private individuals. The plain and undeniable fact is, that, in about fifty-five minutes' mesmerising, this woman, deemed incurable, who for two months had not been able to walk without her crutch, and had been in violent pain, was enabled to walk alone without pain, and so well that even medical observers could not perceive any lameness. One more patient in this hospital was also strongly affected; but I pass over her case, to hurry on to the metropolis of science.

April 21st, at St. George's Hospital, London, I mesmerised two female patients, in the presence of Mr. Brodie, Mr. Smith, of the Coldstream Guards, and some other persons. The effect was hardly perceptible.

April 26th.—I had two patients at my own house, sisters, both epileptic from their childhood, and daughters of an epileptic father. Mary Ann was aged nineteen; Sarah, sixteen. The former had been mesmerised once, on the preceding day; the latter I had never seen before. I mesmerised them both this day, in presence of Dr. Milligan and Mr. Smith; but I shall confine my account to Sarah, whom I mesmerised for the first time. After a very few passes her eyes closed, and in about one minute she was in sound mesmeric sleep. She awoke when I spoke to her, was sickish in her stomach, and had a pain in her head. A few transverse passes removed her headach, but the sickness in her stomach remained. After the operation, Dr. Milligan declared his opinion to be that the somnolency produced upon this patient was the effect of a peculiar influence exercised by me; and that, on account of its suddenness, it could not be the result of any of the usual modes which produce sleep. Mr. Smith coincided in this opinion. I shall presently quote Dr. Milligan upon another occasion.

On the eleventh day of Mary Ann H. and the tenth of Sarah H., May 7th, Mr. Brodie, Dr. Prout, and Capt. Bagnold, were present.

Mary Ann H. appeared to sleep in about four minutes. In twenty minutes I woke her by speaking to her. She had a headach, which a few transverse passes, made with that intention, removed.

Sarah H. had a headach when I began, which a few transverse passes dissipated. Her eyes closed almost instantly, and in about one minute she was fast asleep. While she was in that state, I opened her eyelids, in order to show that the appearance of the eye was different from what it is in waking persons. As these gentlemen were hurried, I awoke both these patients in twenty-two minutes. The opinion of Mr. Brodie, on seeing these experiments, was doubtful as to the reality of Mary Ann's sleep. The following conversation took place respecting her sister. I said, "Let me ask you two questions; but beware of your answers, for it is fair to tell you that I wish to have them for the express purpose of publication: Do you think this girl really and truly slept?" "I do, and very soundly too." "Do you think she went to sleep herself, out of fatigue or ennui, or, in short, by means of what you saw me do?" "Certainly by means of what you did." In a subsequent conversation, Mr. Brodie said that, though he did not doubt that sleep had been brought on by means of what I did, he saw no necessity for supposing the existence of any new agency; for the effect may be accounted for upon the same principle as giddiness, &c. produced by a quick rotatory motion.

Dr. Prout doubted whether Mary Ann really was asleep, but he had no doubt as to Sarah. He was by no means convinced, however, that my action was the cause of her sleep; though he did not immediately see to what other agency it could be ascribed. He added, that the circumstance of these girls having been so often acted upon before makes the subject still more doubtful in his estimation.

The opinion of Capt. Bagnold was, that both patients were asleep in three minutes and a half; and in this belief I perfectly coincided.

The fact is, however, that the appearance of Mary Ann was much more equivocal to persons who never before had witnessed mesmeric phenomena; while it was impossible to hesitate one moment respecting her sister. But let Mary Ann be entirely struck off the list of facts, Sarah still slept; and that she did sleep, is testified by the above highly credible witnesses.

The preceding statement was submitted to Mr. Brodie and to Dr. Prout for their approbation, and the following answers were returned in writing.

16, *Saville row; May* 13, 1829.

"My dear Sir: What I have to say on the subject of the experiments on the two girls which I saw made by you in Jermyn street, is as follows: I have my doubts whether the eldest of these girls really slept. I have no doubt that the other slept very soundly for several minutes, and until you awoke her. I have no doubt also that she slept in consequence of the means employed by you. With such information, however, as I at this moment possess, I see no reason to believe that this girl's sleep may not be explained on principles already known; and I should think that it may be compared to the giddiness which is produced by turning round; or, still better, to the sleep produced by rocking a child.*

"Believe me to be, my dear sir, yours most truly,

"B. C. Brodie."

"*29th May.*

"Dear Sir: I return your statement with some slight alterations, (which made it as given above, relating to Dr. Prout's testimony,) &c. * * I fear, on the whole, my evidence is rather against you; and I confess that I must see much more before I can be satisfied of the reality of mesmerism.

"Most truly yours, N. Prout."

On May 12th, the fifteenth time of Mary Ann, and the fourteenth of Sarah H., Mr. Faraday and Dr. Hargood, both of the Royal Institution, were present.—Sarah H. complained of a pain in her stomach and head, which a few transverse passes removed. I then directed my intention toward producing sleep. After a pass or two her eyelids closed, and in less than two minutes she was asleep. While she was in that state, I raised her left eyelid, and showed the eye under it with the glossy and lifeless appearance which it generally assumes when she is in mesmeric sleep. In thirty minutes I awoke her, calling her by name.

Mary Ann slept in about three minutes, and very soundly. I uncovered her eye also, which was not quite so much affected as her sister's. In thirty minutes I awoke her. She had a violent headach, which a few transverse passes calmed.

Mr. Faraday's opinion was, that there was nothing in these phenomena which a good actor could not play. A circumstance which induced him to doubt the reality of the sleep of Mary Ann, was that she coughed, and put her hand before her mouth, as she might have done being awake. But it is a great error to confound mesmeric sleep with common sleep; for, in the former, many appearances are assumed which are incompatible with the latter. The second person I ever saw in the state of artificial somnambulism walked about the house, and performed many do-

* This is precisely the explanation of mesmeric sleep which we offered in our review of Dupau on Animal Magnetism, in our Journal for November, 1826, p. 469; and we are, of course, confirmed in the opinion we then expressed by finding it supported by Mr. Brodie. In speaking of this phenomenon, we observed "There is here no mystery: the effect might be anticipated. The magnetic fluid is not required. Upon the same principle, a child is lulled to rest by fatiguing its senses with some nursery lullaby, or some gentle and oft-repeated motion."—Editors.

mestic functions while in that state. In natural somnambulism the fact has long been acknowledged.

Dr. Hargood was struck at the promptness with which the pain in Sarah's head and stomach were relieved by the transverse passes made with that intention, before the operation for sleep was begun. He believed both sisters to be soundly asleep, but attributed all the phenomena which he saw to imagination. Both Mr. F. and Dr. H , however, considered these experiments as well worthy of investigation, and expressed their wish that the subject might meet with fair and candid inquiry.

May 16th, I mesmerised Mary Ann for the eighteenth time. Unfortunately, her sister, who was in service, could not come this day. The Marquess of Lansdowne and Dr. Holland were present. In about five minutes the girl was asleep. I opened her eyelid, and showed the state of the eye under it, fixed, and with a dead and glassy appearance.* She was slightly disturbed by this experiment, yet she continued asleep. At the expiration of about twenty minutes I awoke her. I then put the same questions to Dr. Holland as I had put to Mr. Brodie; telling him also that my object was to have his testimony, be it what it might, for publication. "Do you think this girl was asleep?" "I do." "Do you think she went to sleep of herself, out of lassitude, ennui, or by means of what I did to her?" "Certainly I think that, without your means, she would not have slept." I asked the same questions of Lord Lansdowne, and received the same answers. Neither Lord Lansdowne nor Dr. Holland, however, admitted the necessity of any new agency to account for these effects.

This statement was sent to Dr. H. for his approbation, and his answer was as follows:

"Dear Sir: I return the enclosed paper, having nothing to object to the statement respecting the girl whom I saw at your house. I believe that she was asleep, and that she would not have slept but for the means employed by you.

"Most truly yours, H. Holland."

May 19th, the seventeenth sitting of Sarah H. Drs. Babington and B. Babington present.—She slept in two minutes. I opened her eye, and pressed my finger hard upon the eyeball: she did not make the slightest motion. I then put my middle finger into her mouth as far as

* Mr. Mayo, who accidentally saw this passage in its way through the press, observed to us that, in a doubt between genuine and pretended sleep, the state of the pupil is a valuable criterion. In natural sleep, the pupil, according to Mr. Mayo, is always greatly contracted.—Editors.

I could, and stirred it about for more than a minute, endeavouring to stimulate the fauces: she showed not the slightest symptom of feeling any thing from this operation. I then tickled her nose and upper lip with a slip of paper, and put the same slip of paper up her nostril; but she did not manifest the slightest sensibility to the impressions which should have resulted in ordinary cases.

Dr. Babington said that, if long experience of the many impositions practised by the poorer classes in the hospitals had not put him upon his guard, he might be induced to give some credit to these appearances; but he wished to suspend his judgment until he could make some experiments himself, in which he would use every means to guard against deception on their part. I observed to him, that in this case there could be no motive for imposition, as the girl came to me every day from a considerable distance, without a hope of any remuneration but health. My wish, however, being much more to excite active curiosity than to impart supine conviction, most heartily do I hope that many will adopt the same mode of obtaining truth.

This statement was submitted to Dr. B. for his approbation. He confirmed it in writing; adding, that he had not yet had an opportunity of showing it to his son; "but I have no doubt that he will agree with me in finding it quite correct."

Dr. B. Babington has since written, to say that the two girls "did present the usual phenomena of sleep: I may even say, of profound sleep. This did not, however, appear to me to be induced by any new or extraordinary influence, and it seemed that the imagination, aided by the will, exercised a power over the faculties," &c.

On April 27th and 28th, I tried some patients at the Middlesex Infirmary in Great Pulteney street, but with little success.

April 29th, I mesmerised an epileptic boy. In seven minutes his eyes closed, and his head fell back; he did not entirely sleep, however. On coming to himself he felt a trembling, which I soon calmed. Mr. Evans Riadore, of this establishment, was present, but said he saw nothing which could authorize him to admit a mesmeric effect.

April 30th.—Mesmerised the same boy again. Dr. Milligan and Mr. Evans Riadore were present. No somnolency was produced; but in six minutes a tremor ensued, as yesterday, and the boy said he felt a fit coming on. I increased the intensity of the mesmeric action, with the intention of preventing the fit, but without giving him any intimation of my purpose. In three minutes he said he was much better; and in two minutes more he said he was quite well, with the exception of a headach, which a few transverse passes, with an intention to demesmerise him, took away. During the tremor he became very pale, and after it, Mr. Evans Riadore found his pulse slower than before. Dr. Milligan, who had witnessed the first day's mesmerization of Sarah H., reaped new conviction from this patient. Yesterday, Mr. Evans Riadore had said to me, in conversation, that mesmerism seemed so repugnant to his reason, that he could not bring his mind to consider it at all. This day he exclaimed, "I have seen something today, I must confess, and I did not expect it. There certainly is something in all this, and I will try it in this very institution." "Will you sign this," said I, "and let me publish it?" He answered, "I will." To which Dr. Milligan added, "There is nothing which we say to you on this head that you may not publish."

The handsome manner in which these gentlemen avowed their conviction enhances its value. It is not every man who today will admit, even upon experiment, what yesterday he thought repugnant to his reason. Dr. Milligan and Mr. Riadore did sign this report, approved by them.

The experiments which I shall now relate were performed at St. Bartholomew's Hospital. On May 23d, Mr. Earle was kind enough to allow me to accompany him to that establishment. The first patient submitted to trial was an epileptic young man, who at that moment was taking large doses of nitrate of silver. His fits were very bad and frequent. Though to all appearance this was a person likely to be affected by mesmerism, he manifested little susceptibility; so slight is yet the confidence to be placed in any prognostic relating to this unfathomed subject. Being pressed for time, I continued to operate upon this man only eight minutes.

The next patient was a woman afflicted with disease in her bladder. During the first five minutes no effect was manifested. She then said that she felt a fluttering in her inside. I observed to Mr. Earle, in a language which this woman certainly did not comprehend, "This is a mesmeric effect." Mr. Earle smiled doubtingly. "To convince you," continued I, in the same language, "I will now take this effect away." By altering my intention, and demesmerising the patient, without letting her perceive any alteration, I did calm those feelings. Still Mr. Earle doubted. "I will now," said I, "give her those sensations back again." After two minutes' mesmerising, they returned. "I will now take them away again." I did so, and by the same means. Still, however, though Mr. Earle and a student of the hospital, who was present, acknowledged that the results most accurately corresponded with the intentions which I had announced, conviction made but little progress, so extraordinary did the facts appear; and had not good fortune thrown another patient in my way, on whom the effects were still more palpable, my labour at St. Bartholomew's would have been in vain.

This patient was a woman afflicted with iritis, for which she had been largely bled; and she was, moreover, recovering from a severe mercurial course. In less than two minutes' mesmerising, her head fell back, her eyes closed, and a kind of hysterical trance came on. In three minutes she awoke, said she felt hot, then cold, and a shivering ensued, particularly in her knees and thighs. This I stopped in about one minute, by continuing the mesmeric action in this intention, as I had announced to Mr. Earle in a foreign language. I tried the experiment of the piece of paper on her arm, but she felt it very slightly. Touched with the silver pencil-case, my intention being (as in the cases described in a former article) to give her a sensation of heat, she said she felt as if all the warmth of her hand had gone to that spot. I then demesmerised her, as she complained of much uneasiness; and, having made her stand up, I drew my hands down before her from the head to the very soles of her feet, at the distance of three or four inches, for about one minute and a half, with the intention of destroying the preceding effects. She then said that she felt better, and left the room much recovered. She declared that, in her life, she never had experienced any thing like what she had just felt; that she never had an attack of hysteria, epilepsy, or any nervous paroxysm.

This woman showed considerable susceptibility; and, had time permitted me to continue the treatment, I have no doubt that she would have become a remarkable subject. Mr. Earle assured me that he had witnessed sufficient effects to encourage him to continue the experiments on both these women, and recommended them, for that purpose, to two of his pupils who were present, and to whom I gave all the instructions in my power, pointing out to them the works in which the amplest details upon the modes of operating, together with the dangers and advantages of each, are given. These two gentlemen, also, were fully convinced that extraordinary effects had been produced.

These three patients were entirely selected by Mr. Earle, without my influencing his choice in any manner. I had never seen one of them before, and now only in the presence of incredulous witnesses, eager for truth, who granted nothing that was not proved, and who were very fairly watchful to detect illusion or deception; and all can testify that no act or word of mine could, in the remotest degree, have conduced to intimate to those patients what my intentions were. They came into the room with their minds unsophisticated, unprepared for any result, for any impression; yet, as Mr. Earle saw, at the very first pass of my hand, the last patient began to manifest some of the symptoms so often described in every German and French work on the subject as among those which mesmerism produces; and in less than three minutes was violently affected. I must add that, at the time of operating, I was ignorant of the disorders under which the two female patients were labouring.

On the following day the operation was repeated on the third patient, by one of the pupils; and, in about seven minutes, still more violent convulsive effects were produced, and which lasted longer than on the preceding day. From their violence and duration, indeed, Mr. Earle would not permit the experiments on this patient to be carried to a greater extent.

When this statement was submitted to Mr. Earle, he returned the following answer.

*" George street; May 28th.*

" My dear Sir: In reply to your request that I would state my honest opinion of the trials which you made at St. Bartholomew's Hospital, I have no hesitation in saying that, in the first case, no effect was produced; that, in the second, the patient was under considerable alarm, in expectation that she was about to have her bladder examined, and that she said that she felt a fluttering in her inside, which abated for a time, and was reproduced, as you represent, on your repeating the motions of your hands. In the third case a very decided effect was produced; and it was reproduced the following day by my pupil. In making this acknowledgment, however, I am by no means prepared to say that the effects were any thing more than the influence produced upon the mind of an enfeebled patient by the mysterious movement of your arms, and her ignorance of the object of these movements. The circumstance of her erroneous sensations I have frequently observed after syncope.

" You will perceive, from these observations, that I am yet an unbeliever; but I am quite open to conviction, and will certainly repeat the experiments under less doubtful circumstances. Should more ample experience induce me to alter my opinion, you may depend upon hearing from me. Believe me, my dear sir, very truly yours, " HENRY EARLE."

I shall at this moment refrain from making any observations upon the preceding testimonies, and the modes in which the medical gentlemen, who admit the facts, endeavour to explain them. The next article, which will be the concluding one, will contain the few trials which remain to be mentioned, together with a summing-up of the evidence. I must merely remark, that the convulsions of the third patient, when mesmerised by Mr. Earle's pupil, do not in the least surprise me; neither would they have deterred me from pursuing the experiment. I have never met with one case in which such accidents were attended with danger, provided the operator continued to act upon the patient with an intention of arresting their progress. It requires, however, some experience, and the confidence which practice gives, to enable the mesmeriser to preserve his calmness; and a person who witnesses such phenomena for the first time may, in common prudence, be deterred from carrying the operation any further. This experiment proves, too, that mesmerism is not a thing to be trifled with, and that its power of injury is not less than its power to do good. The only result to be regretted in this case would be, if the alarming symptoms manifested by this patient should prevent the prosecution of the inquiry by so candid an observer as Mr. Earle.

